# Detecting geographical clusters of low birth weight and/or preterm birth in Japan

**DOI:** 10.1038/s41598-023-28642-9

**Published:** 2023-01-31

**Authors:** Md. Obaidur Rahman, Daisuke Yoneoka, Yayoi Murano, Takashi Yorifuji, Hiromichi Shoji, Stuart Gilmour, Yoshiko Yamamoto, Erika Ota

**Affiliations:** 1 Department of Global Health Nursing, Graduate School of Nursing Science, St. Luke’s International University, 10-1 Akashi-cho, Chuo-ku, Tokyo, 104-0044 Japan; 2 Department of Pediatrics and Adolescent Medicine, Faculty of medicine, Juntendo University, 2-1-1 Hongo, Bunkyo-ku, Tokyo, 113-8421 Japan; 3 Division of Biostatistics and Bioinformatics, Graduate School of Public Health, St. Luke’s International University, OMURA Susumu & Mieko Memorial, St. Luke’s Center for Clinical Academia, 3-6-2 Tsukiji, Chuo-ku, Tokyo, 104-0045 Japan; 4 Department of Obstetrics and Gynecology, Faculty of medicine, Juntendo University, 2-1-1 Hongo, Bunkyo-ku, Tokyo, 113-8421 Japan; 5 Department of Health Policy, National Center for Child Health and Development, 2-10-1 Okura, Setagaya-ku, Tokyo, 157-8535 Japan; 6 Center for Surveillance, Immunization, and Epidemiologic Research, National Institute of Infectious Diseases, Tokyo, Japan; 7 Tokyo Foundation for Policy Research, Tokyo, Japan

**Keywords:** Environmental sciences, Medical research, Epidemiology, Paediatric research

## Abstract

In Japan, mean birth weight has significantly decreased from 3152 g in 1979 to 3018 g in 2010 and the prevalence of preterm birth (PTB) has risen to 5.7% in the last thirty years. However, the presence and magnitude of geographical differences in low birthweight (LBW) and/or PTB in Japan is not well understood. We implemented spatial analysis to identify localized clusters and hot spots of LBW and/or PTB during 2012–2016. The Japan national birth database was used in this study. A total of 5,041,685 (male: 2,587,415, female: 2,454,270) births were used for spatial analysis using empirical Bayes estimates of the incidence rate of LBW and/or PTB and spatial scan tests to detect hot-spot areas with *p* values calculated from Monte Carlo iterations. The most and second likely clusters were located in two areas: (1) the small islands in south-west Japan (Amami and Okinawa, Relative risk = 1.09–1.67 with *p* < 0.001) and (2) the cities on the base of Mt. Fuji, stretching over three neighboring prefectures of Yamanashi, Shizuoka and Kanagawa (Relative risk = 1.10–1.55 with *p* < 0.001), respectively. We need to optimize the medical resource allocations based on the evidence in geographical clustering of LBW and/or PTB at specific locations in Japan.

## Introduction

Low birth weight (LBW) is defined by the World Health Organization (WHO) as birth weight less than 2500 g (5.5 lb) irrespective of gestational age. LBW is an important global health issue, having both short- and long-term health consequences. LBW newborns who survive have a higher risk of short-term and long-term morbidities, including non-communicable diseases such as diabetes, hypertension and other cardiovascular diseases^1^. LBW is the leading cause of death in under-five children worldwide. It accounts for 80% of all neonatal deaths, of which two-thirds are preterm birth (PTB)^2–6^. Around 15 million PTB and more than 20 million LBW, which is one in seven of all births in the world, were observed globally in 2014 and 2015, respectively^7,8^. In 2012, a Comprehensive Implementation Plan on Maternal, Infant and Young Child Nutrition was introduced by the World Health Assembly and all member states committed to achieve a global nutrition target of a 30% reduction in the number of infants with LBW by 2025, relative a 2012 baseline^9^. Recent estimates found that the global LBW incidence has fallen slightly from 17.5% in 2000 to 14.6% in 2015^7^, while the incidence of PTB has increased from 9.8 to 10.6% over the period^8^. Further, progress toward the target is quite slow in high-income countries such as the UK, Finland, France, Germany, the USA, Australia, and New Zealand^7,8^. To meet the global LBW target by the year of 2025, progress must more than double, and improved measurement and program investment to address the causes of LBW throughout the lifecycle is urgently required^7^.

Despite having an advanced healthcare system, Japan has the second highest prevalence of LBW among the Organization for Economic Cooperation and Development (OECD) countries. In Japan, the prevalence of LBW has increased from 8.6% in 2000 to 9.5% in 2015^7^. The mean birthweight of singleton infants in Japan has significantly decreased from 3152 g in 1979 to 3018 g in 2010^10^. PTB prevalence has also increased to 5.7% in the last thirty years^8^. Several studies reported the trends and maternal risk factors, including parental socio-demographic and economic factors, for LBW and PTB in Japan^10–17^. However, there is a lack of evidence of identifying the geographical clusters of LBW and/or PTB in Japan, even though geographical disparities have been identified in other countries and may play an important role in the persistence of PTB and LBW in developed nations^18–20^. To the best of our knowledge, only one study explored spatial patterns of LBW in a single administrative area of Japan and reported some LBW prevalent clusters^21^. Geographical analysis for identifying geographical clusters of LBW and/or PTB helps policymakers to identify priority areas of action as well as boosting public health promotion policies for addressing health issues in those particular areas. This study explores the spatial variation in LBW and/or PTB across all areas in Japan by identifying clusters with statistically significantly elevated or reduced incidence of these events, using a robust national dataset.

## Methods

### Dataset, exposure and outcomes

Birth certificate data was obtained from the Ministry of Health, Labor and Welfare in Japan vital registration statistics^22^. Based on the Guidelines for Statistics Law promulgated by the Ministry of Internal Affairs, our application documents for the secondary use of the Vital statistics were submitted to the MHLW and approved. Data from January 1st, 2012 to December 31st, 2016 was extracted for our analysis. In addition to the individual’s birthweight, maternal age, residential address and gestational age were extracted. We excluded multiple births to avoid the bias of a higher risk of LBW for multiple births^10^. Every infant was categorized as LBW (birthweight < 2500 g) or normal birthweight (NBW) group (birthweight ≥ 2500 g). In addition, they were also categorized as PTB (gestational age of < 37 weeks) or term birth (gestational age of ≥ 37 weeks). Consequently, the following four groups were considered in this study: LBW, PTB, LBW and term birth (LBW term) and LBW and preterm birth (LBW pre). Since we used national data that the medical profession is required to input at birth, there are essentially no (or at least very small) missing values. If there were missing values, they were dropped.

### Statistical/geographical analysis

Japan was divided into 1902 municipalities at the time of the study, organized within 47 prefecture^23^. Geographical analysis in this study was conducted at the municipality level. Geographical coordinates defined as the centroid of the area (measured by latitude and longitude) were extracted from map data for each municipality (https://nlftp.mlit.go.jp/ksj/jpgis/datalist/KsjTmplt-N03.html). Empirical Bayes estimates of the age-standardized incidence rate (EBSIR) were used to examine how LBW and/or PTB were geographically distributed in Japan during the study period^24,25^. The age-standardization for each municipality was implemented based on the age structure of Japanese population as a reference: the age categories were ≤ 15, (15,20], (20,25], (25,30], (30,35], (35,40], (40,45], (45,50] and ≥ 50. In addition, when no birth was observed among certain age groups in a small municipality, we imputed a very small value (i.e., 0.0001) for calculating the age-standardized value. We first calculated the age-standardized values using a direct method, and then the Empirical Bayes estimates was calculated. The advantage of EBSIR is that it can incorporate the information of the spatial neighborhood areas to smooth the risk of LBW and/or preterm birth toward the local (spatial) neighborhood mean, stabilizing estimates for municipalities with small numbers of births based on updated information from neighboring municipalities^24–26^. The spatial neighborhood was defined as the queen contiguity where spatial neighbors shared at least a common border. The local adjacency matrix was calculated based on the *k*-nearest neighborhood method with *k* = 8.

Spatial clusters were defined following: “a geographically bounded group of occurrences of sufficient size and concentration to be unlikely to have occurred by chance”^27^. Tango’s statistic for general clustering was used to globally test the existence of clusters in Japan^28^. Then, to identify the exact locations of geographical clusters of LBW and/or PTB in Japan, the spatial scan statistic and its associated likelihood-based test proposed by Kulldorff and Nagarwalla (1995) were implemented^29,30^. The test can identify spatial clusters of any size, located anywhere in the study region. Under the assumption of a Poisson distribution for the occurrence of LBW and/or PTB, the most/second/third likely clusters (MLC/SLC/TLC) were defined by maximum likelihood estimation^31^. To obtain the corresponding *p* values, 1000 Monte Carlo (resampling with replacement) iterations were implemented^29,30^. Data management was done by using AWK, SQL and Python and statistical analysis was performed with R version 3.6.1. In this paper, statistical significance was defined as a *p* value less than 0.05. This study was approved by the Ethics Committee at National Center for Child Health and Development (No. 1274). Informed consent is waived by the Ethics Committee at National Center for Child Health and Development. All methods were carried out in accordance with relevant guidelines and regulations.

## Results

### Study samples

**Figure 1:**
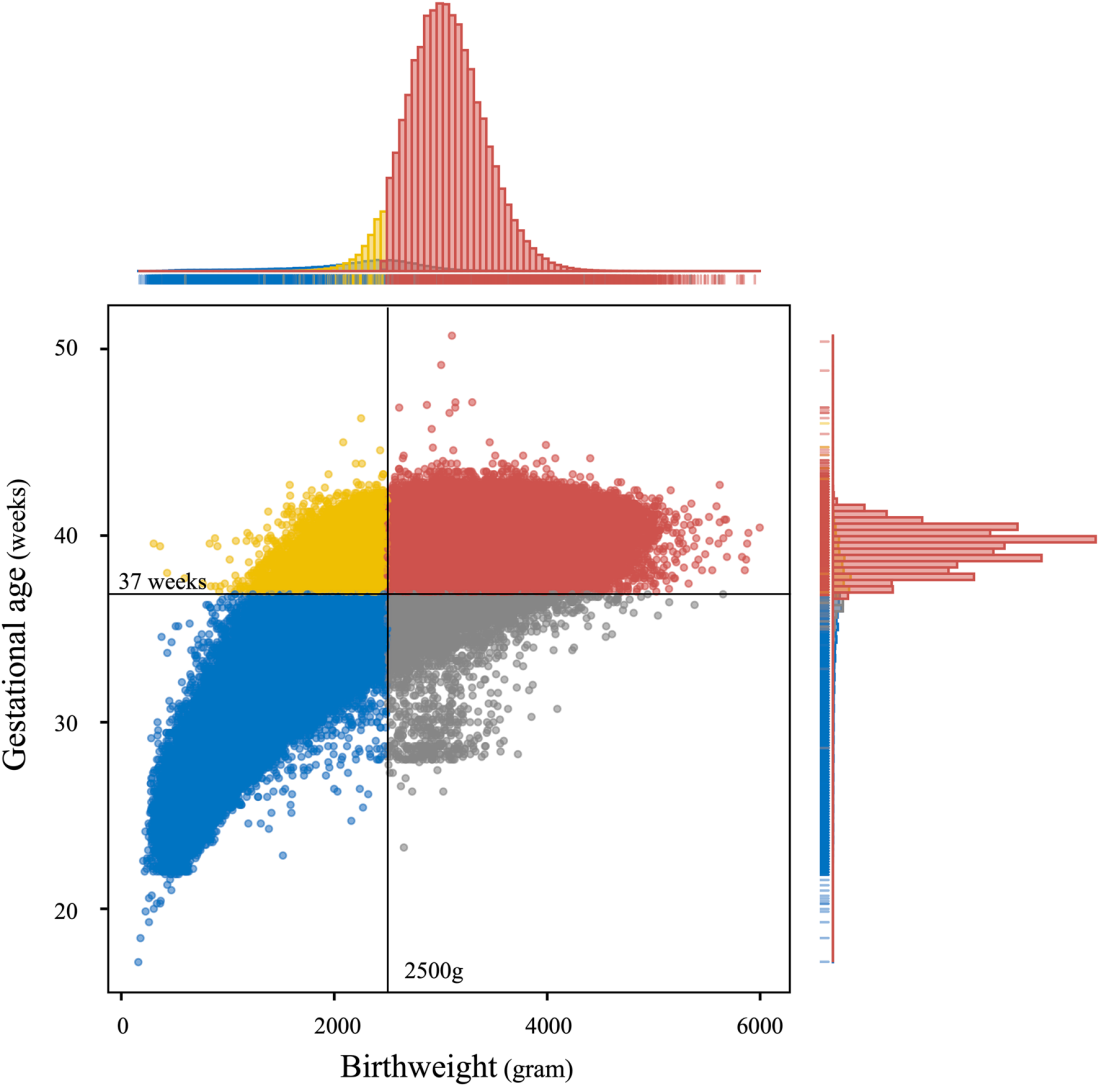
Distribution of LBW and preterm birth in Japan from 2012–2016: Blue (LBW pre), Yellow (LBW term), Gray (Normal and Preterm birth), and Red (Normal and Term birth).

A total of 5,041,685 (male: 2,587,415, female: 2,454,270) births were reported in Japan from 2012 to 2016. Among them, 8.23%, 4.74%, 5.23%, and 3.00% were categorized as LBW, PTB, LBWat term, and LBWbut premature, respectively. The reported proportions of births by maternal age were 2.21%, 76.64% and 21.1% for the age of ≤ 20, (20, 35] and > 35, respectively. The detailed characteristics are shown in Table 1 and Fig. 1 shows the scatter plot of birthweight and gestational age.

**Table 1:** Basic characteristics of LBW, Preterm, and LBWterm, and LBWpre.

	Total		LBW		Preterm		LBWterm		LBWpre	
N	5,041,685		415,076		239,026		263,641		151,435	
	Frequency	%	Frequency	%	Frequency	%	Frequency	%	Frequency	%
Survey year										
2012	1,033,940	20.5	85,627	20.6	49,324	20.6	54,558	20.7	31,069	20.5
2013	1,025,729	20.3	84,861	20.4	49,213	20.6	53,759	20.4	31,102	20.5
2014	1,001,899	19.9	82,750	19.9	47,676	19.9	52,288	19.8	30,462	20.1
2015	1,003,031	19.9	82,338	19.8	46,925	19.6	52,546	19.9	29,792	19.7
2016	977,086	19.4	79,500	19.2	45,888	19.2	50,490	19.2	29,010	19.2
Nationality										
Japanese living in Japan	4,953,308	98.2	410,041	98.8	234,185	98.0	261,223	99.1	148,818	98.3
Foreigners living in Japan	71,418	1.4	4057	1.0	3997	1.7	1882	0.7	2175	1.4
Japanese living abroad	13,405	0.3	633	0.2	579	0.2	348	0.1	285	0.2
Other	3554	0.1	345	0.1	265	0.1	188	0.1	157	0.1
Sex										
Male	2,587,415	51.3	186,727	45.0	137,566	57.6	104,767	39.7	81,960	54.1
Female	2,454,270	48.7	228,349	55.0	101,460	42.4	158,874	60.3	69,475	45.9
Maternal age										
≤ 20	111,256	2.2	10,338	2.5	5949	2.5	6361	2.4	3977	2.6
(20, 35]	3,864,254	76.6	302,886	73.0	168,627	70.5	197,094	74.8	105,792	69.9
> 35	1,066,169	21.1	101,851	24.5	64,449	27.0	60,186	22.8	41,665	27.5
Mean of birthweight (IQR)	3020.1 (2778, 3280)		2187.7 (2124, 2430)		2232.5 (1950, 2630)		2340.7 (2280, 2450)		1921.2 (1656, 2320)	
Mean of gestational age (IQR)	38.8 (38, 40)		36.4 (36, 38)		34.3 (34, 36)		38.1 (37, 39)		33.5 (32, 36)	

*IQR* inter quantile range.

### Geographical distributions

**Figure 2:**
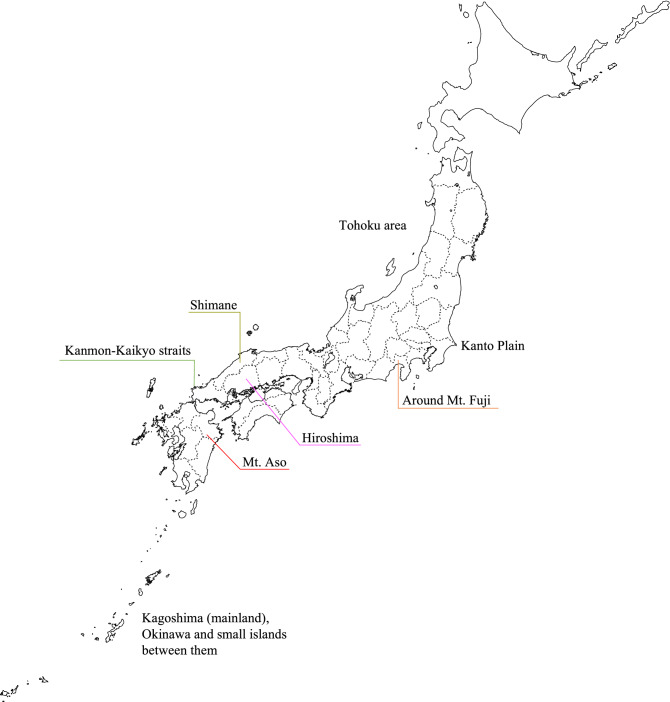
Some key locations in Japan.

**Figure 3:**
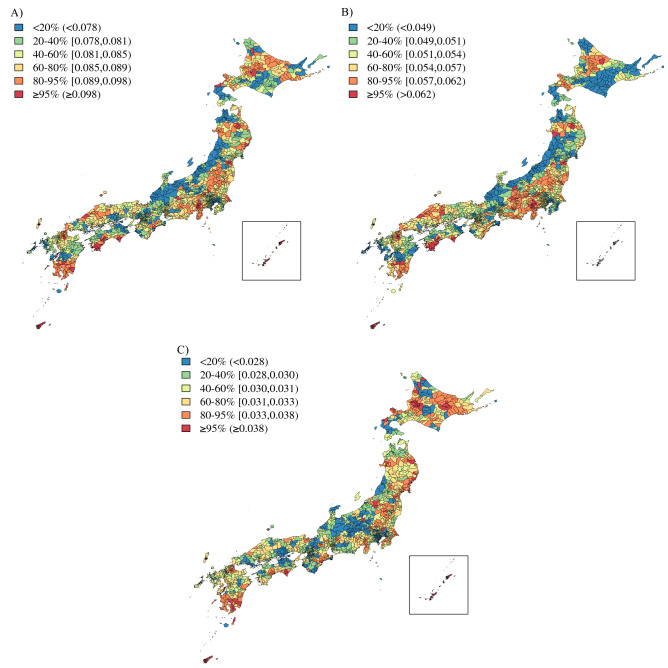
Empirical Bayes estimates of age-adjusted incidence rate of LBW (**A**, left), LBW term (**B**, center) and LBW pre (**C**, right) in Japan: color scale indicates quantile.

**Figure 4:**
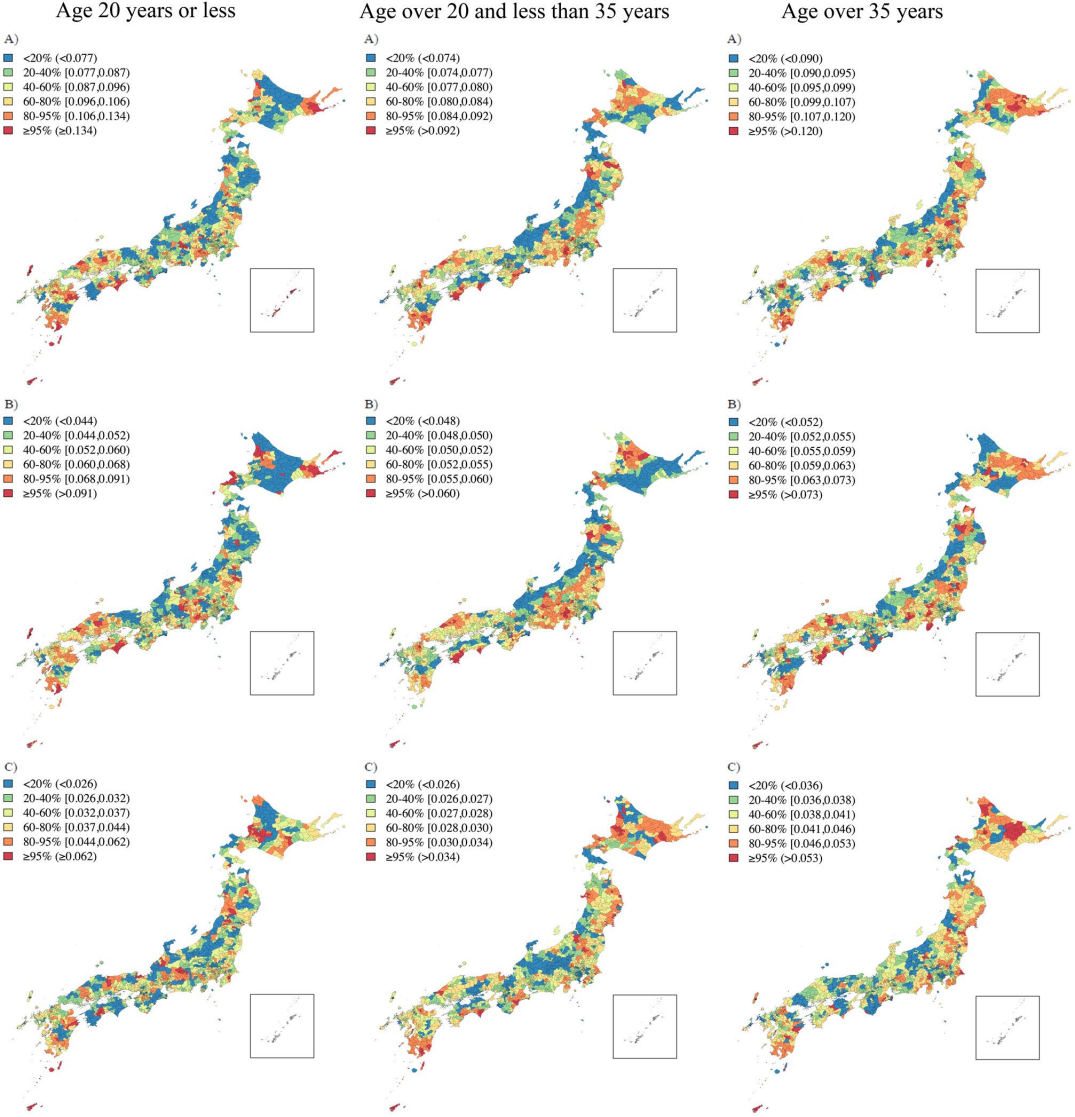
Empirical Bayes estimates of incidence rate of LBW (**A**, upper), LBW term (**B**, middle) and LBW pre (**C**, lower) separately by age groups (20 years or less, 20 and less than 35 years, and over 35 years) in Japan: color scale indicates quantile. This map was created using R version 3.6.1 (https://cran.r-project.org/) and zipangu package.

Figure 2 shows the Japanese map and indicates some key locations. The geographical distributions of the EBSIR of LBW and/or PTB were estimated for all women (Fig. 3) and separately by age groups (Fig. 4). The test of the homogeneity of the relative risk measured by Tango’s statistics showed that there was a significant heterogeneity of risk in all groups (*p* < 0.001 for all sub-groups). Tables 2, 3 and 4 and Supplemental Figs. 1, 2 and 3 show the approximate locations of MLC/SLC/TLC of LBW and/or PTB. As shown in Tables 2, 3, and 4, the most frequently detected area in several sub-groups was an archipelago in the south-west part of Japan (Area 1: A1, A1_1, A2, A2_2, A3, A3_1, A3_2, A3_3, B1_2, and C2_2. Relative risk = 1.09–1.67 with *p* < 0.001). The second most frequently detected area was highland cities around the north-east part of Mt. Fuji (Area 2: A2_2, B1, B1_2, B1_3, B2, B2_1, B2_3, and C3_3. Relative risk = 1.10–1.55 with *p* < 0.001). The third frequently detected area was cities around the Kanmon-Kaikyo strait that separates the main island and Kyushu island (Area 3: B2_2, C1, C1_2, C2, C3, and C3_2. Relative risk = 1.08–1.32 with *p* < 0.001). It is noteworthy that we observed a distinctive difference in the locations of the clusters between LBW term and LBW pre: LBW infants at term were likely to be clustered around the north-east part of Mt. Fuji (A2_2, B2_1, B2_3, and C2, which were partially detected in Area 1), while LBW and premature infants were likely to be clustered around Tohoku or the north part of the Kanto Plain (B3, B3_2, B3_3, and C3_1).

**Table 2:** Geographical clusters of LBW in Japan.

Cluster	Cluster location	Number of municipalities	Expected cases	Observed cases	Relative risk	*p* value
Total (n = 415,076)						
Most likely cluster	A1: Kagoshima (mainland), Okinawa and small islands between them	79	12,672.10	15,174	1.20	< 0.001
Second likely cluster	B1: Around Mt. Fuji	73	11,743.66	12,890	1.10	< 0.001
Third likely cluster	C1: Kanmon-Kaikyo Straits between Honshu and Kyushu	50	9009.31	9850	1.09	< 0.001
Maternal age ≤ 20 (n = 10,338)						
Most likely cluster	A1_1: Small islands in Kagoshima and Okinawa	44	319.55	466	1.46	< 0.001
Second likely cluster	B1_1: East of Mt. Aso	17	122.65	165	1.35	< 0.001
Third likely cluster	C1_1: West of Hiroshima and Shimane	5	49.34	74	1.50	< 0.001
Maternal age (20, 35] (n = 302,886)						
Most likely cluster	A1_2: Kagoshima (mainland), Okinawa and small islands between them	77	9182.24	10,891	1.19	< 0.001
Second likely cluster	B1_2: Around Mt. Fuji	60	6324.67	6988	1.10	< 0.001
Third likely cluster	C1_2: Kanmon-Kaikyo Straits between Honshu and Kyushu	50	6806.81	7384	1.08	< 0.001
Maternal age > 35 (n = 101,851)						
Most likely cluster	A1_3: Kagoshima (mainland), Okinawa and small islands between them	92	3038.13	3670	1.21	< 0.001
Second likely cluster	B1_3: Around Mt. Fuji	79	2714.57	3119	1.15	< 0.001
Third likely cluster	C1_3: North part of Kanto Plain: Gunma, Tochigi and Fukushima	65	2675.69	3032	1.13	< 0.001

**Table 3:** Geographical clusters of LBWterm in Japan.

Cluster	Cluster location	Number of municipalities	Expected cases	Observed cases	Relative risk	*p* value
Total (n = 263,641)						
Most likely cluster	A2: Kagoshima (mainland), Okinawa and small islands between them	77	8009.59	8936	1.12	< 0.001
Second likely cluster	B2: Around Mt. Fuji	87	7581.08	8483	1.12	< 0.001
Third likely cluster	C2: Kanmon Straits between Honshu and Kyushu	47	5491.06	6003	1.09	< 0.001
Maternal age ≤ 20 (n = 6361)						
Most likely cluster	A2_1: Small islands in Kagoshima and Okinawa	44	196.60	261	1.33	< 0.001
Second likely cluster	B2_1: Around Mt. Fuji	18	68.49	106	1.55	< 0.001
Third likely cluster	C2_1: Hiroshima and Shimane	51	184.88	230	1.24	< 0.001
Maternal age (20, 35] (n = 197,094)						
Most likely cluster	A2_2: Around Mt. Fuji	71	4367.40	4952	1.13	< 0.001
Second likely cluster	B2_2: Kanmon Straits between Honshu and Kyushu	5	746.81	985	1.32	< 0.001
Third likely cluster	C2_2: Small islands in Okinawa	47	4237.07	4617	1.09	< 0.001
Maternal age > 35 (n = 60,186)						
Most likely cluster	A2_3: North part of Kanto Plain: Gunma, Tochigi and Fukushima	64	1307.07	1524	1.17	< 0.001
Second likely cluster	B2_3: Around Mt. Fuji	45	1485.61	1711	1.15	< 0.001
Third likely cluster	C2_3: East of Kagoshima mainland	28	527.07	648	1.23	< 0.001

**Table 4:** Geographical clusters of LBWpre in Japan.

Cluster	Cluster location	Number of municipalities	Expected cases	Observed cases	Relative risk	*p* value
Total (n = 151,435)						
Most likely cluster	A3: Kagoshima (mainland), Okinawa and small islands between them	78	5066.74	6726	1.33	< 0.001
Second likely cluster	B3: Center of Tohoku area	89	3939.28	4339	1.10	< 0.001
Third likely cluster	C3: Kanmon-Kaikyo Straits between Honshu and Kyushu	36	2266.57	2579	1.14	< 0.001
Maternal age ≤ 20 (n = 3977)						
Most likely cluster	A3_1: Small islands in Kagoshima and Okinawa	47	123.41	206	1.67	< 0.001
Second likely cluster	B3_1: Center of Hiroshima	14	53.88	78	1.45	< 0.001
Third likely cluster	C3_1: Northwest of Tokyo	3	13.05	28	2.15	< 0.001
Maternal age (20, 35] (n = 105,792)						
Most likely cluster	A3_2: Kagoshima (mainland), Okinawa and small islands between them	77	3539.77	4672	1.32	< 0.001
Second likely cluster	B3_2: Center of Tohoku area	81	2904.96	3155	1.09	< 0.001
Third likely cluster	C3_2: Kanmon-Kaikyo Straits between Honshu and Kyushu	38	1687.92	1883	1.12	< 0.001
Maternal age > 35 (n = 41,655)						
Most likely cluster	A3_3: Kagoshima (mainland), Okinawa and small islands between them	91	1251.77	1665	1.33	< 0.001
Second likely cluster	B3_3: North part of Kanto Plain: Gunma, Tochigi and Fukushima	81	1064.98	1260	1.18	< 0.001
Third likely cluster	C3_3: Around Mt. Fuji	42	653.15	804	1.23	< 0.001

## Discussion

The study revealed the geographical distribution of LBW and/or PTB in Japan from 2012 to 2016 using population-based data. To the best of our knowledge, this is the first study to identify geographical clusters of LBW and PTB among Japanese singleton live-birth infants using spatial analytical techniques at the municipality level. In this study, we identified several characteristic areas, generally called ‘hot spots’, in an archipelago of small islands in south-west Japan (Amami and Okinawa), and a cluster of cities on the base of Mt. Fuji, stretching over three neighboring prefectures of Yamanashi, Shizuoka and Kanagawa. Although we identified more than two geographical clusters, we confine our discussion to these two areas because they were frequently identified in several sub-groups (Tables 2, 3, 4). In Area 1, both term and premature low birth weight incidence were highly clustered. It is well known that Okinawa prefecture, which is the center of Area 1, has had an incidence rate of LBW that is consistently 20% higher than the rest of Japan since the 1970s^32^. Previous studies have reported that residents in Area 1 have relatively higher values of risks factors for poor birth outcomes, compared with other areas, such as high smoking rates among pregnant women^33^ and low parental socioeconomic status (SES), including low household income^34^. Area 1 has the lowest percentage of students attending college or university in Japan, and these low education levels might have a significant impact on the incidence of LBW^35^. Finally, Area 1 is located far from the main islands of Japan. Residents of Area 1 are genetically isolated from the major island of Japan^36^, and thus one of the reasons for the regional difference might be a genetic issue.

In contrast, in Area 2 LBW was only highly clustered among pregnancies brought to term. Residents in Area 2, especially the eastern part of Shizuoka prefecture, which is the center of Area 2, have limited access to necessary antenatal care: the number of obstetricians and gynecologists here is relatively small (7.4 in 2012 compared to a Japanese average of 8.6), the number of hospitals or clinics for delivery has reduced from 141 to 93 between 1995 and 2010^37^. Further, there is a military training ground of the Japan Ground Self-Defense Force, which is the largest training ground in the main islands of Japan. It implicates that noise and vibration from the military ground and air crafts might influence maternal health^38–40^. Additionally, Modzelewska et al*.* (2019) and Okubo et al. (2015) provided the association between green tea intake, which contains high caffeine, and poor birth outcomes (especially PTB)^41,42^. Green tea is also one of the products with high pesticide residue contain. There is a report that infants with low birth weight have higher contain of pesticide^43^. It is well known that residents in Area 2 have high green tea intake in Japan. The high intake of green tea might be possible epidemiological explanation for Area 2 although there was no available data about the intake of green tea in pregnant women. Our study is the first evidence that Area 2 is a geographically clustered area of LBW term. However, since our discussion here still includes speculations and not based on the specific data, further research will be needed to identify the characteristic risk factors specific to this area.

The present study is limited by the unavailability of information regarding obstetric, genetic, demographic, cultural, and socioeconomic factors of the parents and their children. Although we used a population-based birth certificate dataset that includes all live births in Japan during the study period, the number of available variables in the dataset was limited to residential addresses, the place of birth, birthweight, sex of infant, maternal age, and gestational age at birth. Therefore, further examination would be required to identify the potential risk factors and predictors that can explain the geographical clusters of LBW and/or PTB in Japan. To address this, we are collecting more detailed information such as maternal BMI, smoking status, alcohol consumption, pregnancy complication, family history, and the quality of hospitals as part of an ongoing study into geographical clustering of birth outcomes. Identification of further risk factors and predictors for the geographical clusters could encourage the future development of effective strategies to promote the prevention of LBW and/or PTB in Japan. In addition, this study excluded multiple births, which are high-risk group and requires special attention. Since these births have different pathology, they are excluded from this study, but the current geographical distribution should be also examined carefully to guarantee the optimal allocation of medical resources. It is our ongoing study.

In conclusion, this spatial analysis identified several geographical clusters of term or preterm low birthweight in Japan during the year of 2012–2016. The results can provide a new insight for future genetic, environmental and epidemiological studies on LBW in Japan. It is important to carefully continue monitor the detected areas to ensure that they can receive all the benefits of Japan’s modern health system, and help infants born here to achieve the same health outcomes as the rest of Japan. Moreover, this study showed that even in Japan, a (relatively) homogeneous country, there exist geographical clusters of low birthweight. In addition to standard measures targeting known risk factors of LBW, we suggest that geographical factors should be assessed more in the future.

## Supplementary Information

Supplementary Information.

## Data Availability

The datasets used and/or analyzed during the current study available from the corresponding author on reasonable request.
