# Supplementary material for: Detecting geographical clusters of low birth weight and/or preterm birth in Japan
Source: Sci Rep. 2023 Jan 31;13:1788. doi: 10.1038/s41598-023-28642-9 (PMC9889813; doi:10.1038/s41598-023-28642-9)

Supplementary figures:
Approximate locations of MLC/SLC/TLC. Data is shown in whole age group (left), under 20, between 20 and 35, and over 35, separately. The upper panels show results of LBW, the middle panels LBW term, and the lower panels LBW pre.

Supp. Figure 1.

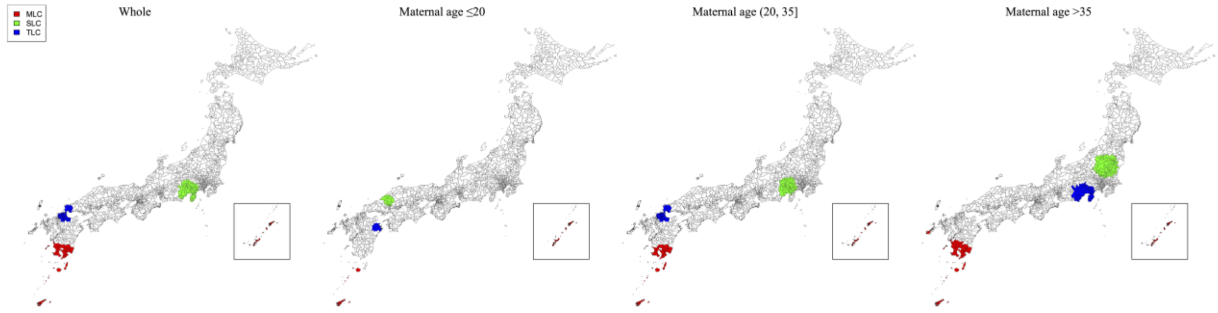

Supp. Figure 2.

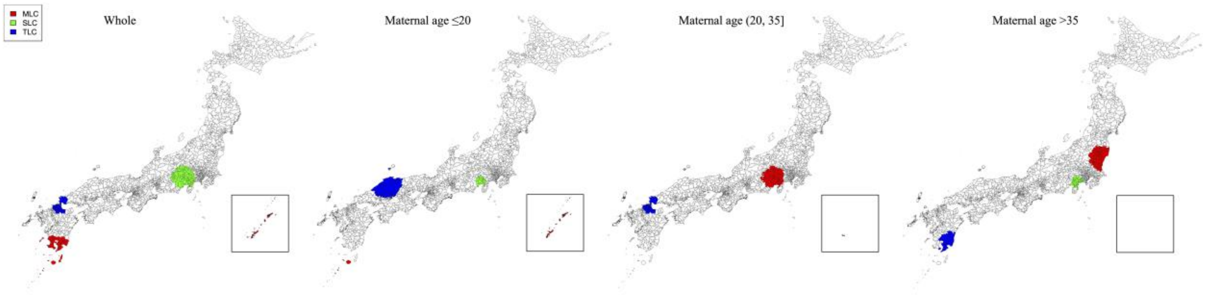

Supp. Figure 3.

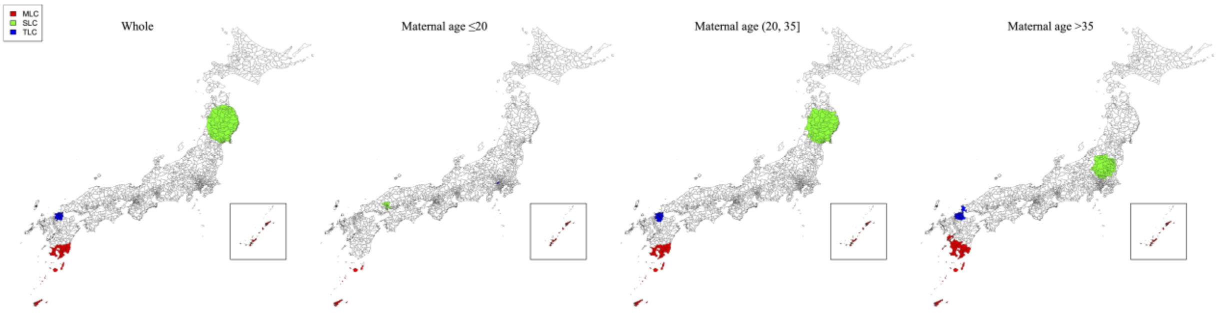

Supplement: Supplementary file 1 — Supplementary Information. [file 41598_2023_28642_MOESM1_ESM.pdf]
